# The Benefit of Cross-Modal Reorganization on Speech Perception in Pediatric Cochlear Implant Recipients Revealed Using Functional Near-Infrared Spectroscopy

**DOI:** 10.3389/fnhum.2020.00308

**Published:** 2020-08-14

**Authors:** Faizah Mushtaq, Ian M. Wiggins, Pádraig T. Kitterick, Carly A. Anderson, Douglas E. H. Hartley

**Affiliations:** ^1^National Institute for Health Research Nottingham Biomedical Research Centre, Nottingham, United Kingdom; ^2^Hearing Sciences, Division of Clinical Neuroscience, School of Medicine, University of Nottingham, Nottingham, United Kingdom; ^3^Nottingham University Hospitals NHS Trust, Nottingham, United Kingdom

**Keywords:** cochlear implantation, neuroimaging, cross-modal plasticity, temporal cortex, auditory processing, visual speech, hearing loss, language

## Abstract

Cochlear implants (CIs) are the most successful treatment for severe-to-profound deafness in children. However, speech outcomes with a CI often lag behind those of normally-hearing children. Some authors have attributed these deficits to the takeover of the auditory temporal cortex by vision following deafness, which has prompted some clinicians to discourage the rehabilitation of pediatric CI recipients using visual speech. We studied this cross-modal activity in the temporal cortex, along with responses to auditory speech and non-speech stimuli, in experienced CI users and normally-hearing controls of school-age, using functional near-infrared spectroscopy. Strikingly, CI users displayed significantly greater cortical responses to visual speech, compared with controls. Importantly, in the same regions, the processing of auditory speech, compared with non-speech stimuli, did not significantly differ between the groups. This suggests that visual and auditory speech are processed synergistically in the temporal cortex of children with CIs, and they should be encouraged, rather than discouraged, to use visual speech.

## Introduction

A cochlear implant (CI) is a neuroprosthetic device that is surgically implanted to partially restore the sensation of hearing in individuals with severe-to-profound deafness and is the therapy of choice for children with congenital and early-onset deafness. Although most CI recipients perform very well with their CIs, speech skills are worse in CI children, compared with their age-matched peers (Holt and Svirsky, 2008; Murphy et al., 2011). It has been shown in adult CI users that cortical responsiveness to visual and auditory stimulation is related to behavioral measures of speech performance (e.g., Lee et al., 2001, 2007; Green et al., 2005; Deshpande et al., 2016; Olds et al., 2016; Anderson et al., 2017, 2019; Zhou et al., 2018). Therefore, cortical correlates of speech perception can offer an insight into the factors influencing speech recognition following cochlear implantation. Although this has been an area of clinical and research interest for some time, insufficient work has been conducted with pediatric volunteers.

The purpose of this study was to explore the relationship between speech understanding and cortical responses in children with CIs and normally-hearing (NH) subjects. These were measured using functional near-infrared spectroscopy (fNIRS), a CI-safe, and child-friendly optical neuroimaging technique (Sevy et al., 2010; Quaresima et al., 2012; Saliba et al., 2016; Harrison and Hartley, 2019). Specifically, in the temporal cortex, we examined three different factors that may correlate with speech perception in CI recipients: responsiveness to visual speech, auditory speech, and non-speech sounds.

A prolonged absence of auditory stimulation is associated with heightened sensitivity to visual stimuli observed in auditory brain regions (e.g., Finney et al., 2001). In CI users, this cross-modal plasticity is typically correlated with poor speech outcomes (Lee et al., 2001; Sandmann et al., 2012; Zhou et al., 2018), frequently attributed to the reduction in auditory cortex ability to process auditory input. Conversely, there is also evidence suggesting a potentially adaptive effect of cortical reorganization in auditory brain regions, with visual input facilitating auditory speech recognition, particularly in cases of post-lingual deafness (Anderson et al., 2017). These contrasting findings suggest that the relationship between the cross-modal recruitment of auditory brain regions by visual stimuli and speech recognition is not well understood and requires further exploration, especially since this factor appears to contribute to CI outcome. Much of the focus of this area of research has been on adult CI listeners and there is a scarcity of similar data in the pediatric implanted population using functional neuroimaging, including fNIRS recordings, with the first fNIRS study with pediatric CI recipients published only a decade ago by Sevy et al. (2010). Furthermore, the influence of (top-down) cross-modal reorganization in adults is likely to play a substantially different role in comparison to the pre-lingually deaf pediatric population (Buckley and Tobey, 2011), when plasticity is sensory-driven (bottom-up; Kral and Eggermont, 2007; Sharma et al., 2007; Campbell and Sharma, 2014). Specifically, during early auditory development, stimulus-dependent pruning of exuberant neural connections is driven by sensory input (Quartz and Sejnowski, 1998; Sharma et al., 2007). When congenital or early-onset deafness limits auditory input, dendritic arborization is disrupted and stimulation deprived pathways in the auditory cortex are left unpruned, providing an opportunity for other sensory modalities to utilize auditory brain areas (Quartz and Sejnowski, 1998; Sharma et al., 2007). After auditory stimulation is later introduced in the form of electrical stimulation *via* a CI, the newly acquired sense is then required to compete for cortical resources, which can be problematic if cortical reorganization has already become “set” (Sharma et al., 2007); perhaps one of the reasons why early implantation is so closely related with better speech outcomes (Robbins et al., 2004; Svirsky et al., 2004; Kral, 2009; Tajudeen et al., 2010). Additionally, animal models have revealed atypical activation patterns and desynchronization of activity between layers in auditory cortices of congenitally deaf cats compared to those with normal hearing (Kral et al., 2002, 2005; Kral and Tillein, 2006; Sharma et al., 2007). Subsequent functional decoupling of primary cortex from higher-order auditory cortex observed following cochlear implantation may also indicate an opportunity for other senses (e.g., vision) to dominate in an auditory cortex deprived of auditory input for an extended period (Kral et al., 2000, 2002, 2005; Kral and Tillein, 2006; Sharma et al., 2007).

In this study, we aimed to examine the influence of cross-modal plasticity on speech understanding in children with CIs (and a group of NH controls). Assessments of low-level visual motion suggest that cross-modal reorganization may be indicative of early auditory deprivation in pediatric listeners and associated with reduced speech recognition (Campbell and Sharma, 2016). Subsequently, we hypothesized that pediatric CI users would, as a consequence of early auditory deprivation, elicit stronger responses to visual speech in auditory brain regions compared with NH controls (Campbell and Sharma, 2016). Furthermore, to enable a comprehensive examination of the effects of temporal cortex cross-modal reorganization, we also investigated cortical responses to normal, auditory speech (without corresponding visual speech). We examined whether the recruitment of auditory regions by visual stimuli occurred at the expense of sensitivity to auditory input. We hypothesized that NH listeners would elicit stronger responses to clear auditory speech than CI users (Olds et al., 2016) to reflect retained auditory processing specialization of the auditory cortex.

As well as investigating responses to auditory and visual speech, we also included two auditory non-speech conditions in the paradigm to examine auditory processing abilities of two specific features of speech [intelligibility and amplitude modulation (AM)] in more detail, including any relationship with speech perception ability. Specifically, since the perception of speech requires the brain to extract linguistic information from acoustic speech signals (Purves et al., 2004), it could be speculated that in some CI users, poor speech perception is a reflection of reduced cognitive ability to derive meaning from otherwise well-transmitted auditory input (i.e., insufficient higher-level auditory processing ability). To examine this hypothesis, cortical responses to intelligible (i.e., auditory speech) and unintelligible (i.e., auditory non-speech) stimuli can be compared, as done previously in both adult (Pollonini et al., 2014; Olds et al., 2016; Lawrence et al., 2018) and pediatric (Mushtaq et al., 2019) listeners using fNIRS. Consistent with findings from these studies, in which greater cortical activation was observed with increased speech intelligibility (Pollonini et al., 2014; Olds et al., 2016; Lawrence et al., 2018), it was hypothesized that intelligible speech (i.e., our normal auditory speech condition) would elicit stronger responses in temporal brain regions of NH listeners, compared with CI users, and that the contrast between cortical responses to the intelligible and unintelligible stimuli will be greatest amongst NH control children. This is based on the assumption that individuals with NH have more refined higher-level auditory processing abilities than CI users, who may struggle to discriminate speech from the CI signal received by the brain (Olds et al., 2016).

Importantly, for the brain to extract linguistic information from speech, the CI must effectively convey acoustic information to the brain. Speech signals are very complex and contain many important acoustic features, one of which is amplitude envelope modulation. With speech stimuli being so highly modulated and numerous features of speech being temporally-defined (e.g., rhythm, stress, and intonation; Rosen, 1992), an alternative approach to investigate the ability of auditory brain regions to recognize speech is to examine cortical sensitivity to modulated and unmodulated auditory stimuli. It is known that CI listeners can make considerable use of AMs to assist with speech recognition (Shannon et al., 1995), as evidenced by some remarkable early studies that showed implant recipients could obtain a degree of speech understanding with only a single channel device (Tyler, 1988; Rosen et al., 1989; Rosen, 1992). This demonstrates that a large proportion of the information necessary for successful speech recognition is associated with the modulating envelope of speech signals (De Ruiter et al., 2015; Dimitrijevic et al., 2016; Han and Dimitrijevic, 2020). Therefore, it is plausible that poor speech performance amongst CI users could result from inadequate transmission of the AM signal from the CI to the brain. In order to explore this idea, we investigated the relationship between fNIRS responses to modulated vs. unmodulated signals. It was hypothesized that NH children would elicit stronger cortical responses to modulated than unmodulated stimuli in temporal brain regions whereas smaller differences would be observed in response to the two stimuli amongst CI users. This would suggest that low-level auditory processing is inadequate in these children, preventing the detection of important AM differences.

## Materials and Methods

### Subjects

Twenty children with NH (mean age 9.5 years; age range 6–12 years; seven males) and 19 CI users (mean age 8.4 years; age range 6–11 years; 12 males) participated in the study. This sample size was based on previous fNIRS studies from our laboratory and elsewhere that involved adult CI recipients using comparable stimuli (e.g., Anderson et al., 2017, 2019; Zhou et al., 2018). All CI users wore bilateral CIs, with the duration of CI use (calculated from the date of CI activation to experiment date) ranging from 29 to 123 months (mean duration 76.8 months). CI users primarily had congenital or early-onset deafness caused by meningitis (three subjects), cytomegalovirus (one subject), Waardenburg syndrome (one subject), connexin 26/30 mutations (two subjects), enlarged vestibular aqueducts (three subjects) and an unspecified genetic cause in one subject. The etiology of hearing loss was unknown for eight subjects. The mean age at CI activation was 27.4 months (range 10–86 months). Details of each CI user’s age at diagnosis and activation along with the duration of CI experience, age, and gender are shown in Table 1. The majority of NH subjects were recruited through online adverts. CI users were chiefly recruited from three pediatric CI clinics in the United Kingdom. These were the Nottingham Auditory Implant Programme, the Midlands Hearing Implant Programme, and the Richard Ramsden Centre for Hearing Implants. All participants were native English speakers with no neurocognitive or motor impairments and with normal or corrected-to-normal vision. All NH participants had no known hearing problems and passed a pure tone audiometry air-conduction hearing screen performed at 0.5, 1, 2, and 4 kHz at 20 dB HL in both ears [test adapted from the British Society of Audiology (British Society of Audiology, 2011)]. The Wechsler Abbreviated Scale of Intelligence—Second Edition (WASI-II; Wechsler, 2011; McCrimmon and Smith, 2012) was administered to assess intelligence. The age-corrected group average intelligence quotient (IQ), derived from the two non-verbal WASI-II subsets (block design and matrix reasoning), was ranked at the 54th percentile (range 8th to 96th percentile) for the NH participants and the 31st percentile (range 2nd to 99th percentile) for the CI group. Handedness was measured using a motor-speech laterality questionnaire by Flowers and Hudson (2013), with sixteen NH children and eighteen CI users assessed as right-handed. Written informed consent was acquired from the accompanying parents, guardians, or relatives of all participants. Verbal assent was obtained from each participant. The study was approved by the University of Nottingham and the East Midlands—Leicester Central Research Ethics Committee.

**Table 1:** Summary of each Cochlear implant (CI) user’s age at diagnosis and CI activation, duration of CI experience, age, and gender (*N* = 19).

Participant ID	Age at diagnosis (months)	Age at activation (months)	Duration of CI experience (months)	Age (years)	Gender
CI_01	Unknown	42	92	11	Male
CI_02	At birth	24	55	6	Female
CI_03	At birth	13	60	6	Male
CI_04	At birth	11	85	8	Female
CI_05	18	20	79	8	Female
CI_06	Unknown	37	78	9	Male
CI_07	28	31	108	11	Female
CI_08	At birth	10	76	7	Female
CI_09	At birth	24	87	9	Female
CI_10	30	55	51	8	Male
CI_11	At birth	86	29	9	Male
CI_12	Unknown	49	59	9	Female
CI_13	At birth	19	81	8	Male
CI_14	10	15	82	8	Male
CI_15	Unknown	16	55	6	Male
CI_16	At birth	10	123	11	Male
CI_17	At birth	26	111	11	Male
CI_18	At birth	15	56	6	Male
CI_19	1	18	92	9	Male
Average		27.4	76.8	8.4	

*Participant identification codes are shown in column one followed by the age of participants when parents report they received a hearing loss diagnosis. The age at activation in months is displayed in column three, which was calculated by subtracting the date of birth from the date of CI activation. The fourth column shows the duration of CI experience, which was calculated by subtracting the date of CI activation from the experiment date. The fifth and sixth columns show each CI participant’s age and gender, respectively. Based on medical history information received from clinicians, eighteen children underwent bilateral implantation simultaneously with only one case of sequential implantation. For children with different activation dates for each CI, the earlier activation date was used for the calculations. For children who underwent re-implantation, their initial and earliest activation date was used for the calculations*.

### Experimental Procedure

#### Speech Perception Test

All participants completed a two-part behavioral speech test to measure speech perception ability. During each part of the test, participants were presented with 16 Bamford-Kowal-Bench (BKB) sentences recited by a male speaker (Bench et al., 1979). BKB sentences are frequently used in audiology and CI departments in the United Kingdom to assess CI outcome and speech performance. Twenty lists containing 16 sentences each were available for this test with one list randomly chosen for each half of the task. Subjects were instructed to listen carefully to the sentences and repeat each one back to the experimenter who then scored the subject against pre-determined keywords. An example sentence with the keywords underlined is: the house had nine rooms. In the first part of the test, sentences were presented in silence and for the second part, they were presented in noise (+10 dB signal-to-noise ratio) to prevent ceiling effects. Note that the background sound was a steady noise with the same long-term average speech spectrum as the target sentences. There were 50 keywords for each part of the test (100 keywords in total).

#### Main fNIRS Task

Participants were presented with a visual speech condition and three auditory conditions in an event-related format. The first auditory stimulus was normal, auditory speech that had not undergone any additional processing and is both modulated and intelligible. Signal-correlated noise (SCN) formed the second auditory condition. SCN is a non-speech signal which is modulated but is unintelligible, and has been used previously in language studies involving neuroimaging (e.g., Stoppelman et al., 2013; Brown et al., 2014; Mushtaq et al., 2019). The third auditory stimulus was steady speech-shaped noise (SSSN), an unmodulated equivalent of SCN. SSSN has also been used numerously in previous speech studies (including those involving hearing-impaired individuals), often as a speech masker (e.g., Oba et al., 2011; Stone et al., 2011; Hall et al., 2012; Porter et al., 2018). Since both SCN and SSSN are unintelligible, intelligibility was not a confounding factor when assessing the effect of modulation. Likewise, modulation was not a confounding factor when assessing the effect of intelligibility as both auditory speech and SCN are modulated. A summary of these characteristics is shown in Table 2.

**Table 2:** Intelligibility and modulation status of each auditory stimulation condition.

	Auditory speech	SCN	SSSN
Intelligible	Yes	No	No
Modulated	Yes	Yes	No

*Auditory speech is both intelligible and modulated whereas steady speech-shaped noise (SSSN) is neither. Signal-correlated noise (SCN) is modulated but unintelligible*.

Eighteen sentences (Hall et al., 2005) were presented randomly per condition, including a muted set to form a silent baseline processing condition. Each sentence lasted approximately 2.97 s on average (range 2.50 s to 3.69 s). Stimulus onset asynchrony (the time between the onset of each sentence) was randomly varied from 4 s to 7 s as the efficiency of event-related studies are enhanced by jittering the stimulus onset asynchrony across trials (Dale, 1999).

**Figure 1:**
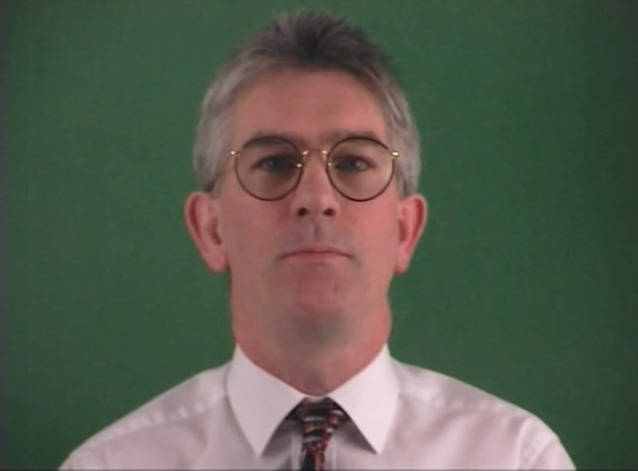
Frame taken from a visual speech stimulus. This picture shows the speaker participants watched during the visual speech trials. No auditory stimulation was presented during these trials as the speaker was muted. The subjects were instructed to focus their attention on the speaker’s mouth (hence were lip-reading). When visual stimuli (or reward stars) were not displayed, a plain dark green background matching the dark green background in this picture was shown with a small fixation cross located at the approximate position of the speaker’s mouth.

Although cortical responses to the auditory stimuli could be measured even with passive listening, subjects needed to keep their visual attention focused on visual stimuli for cortical measurements to be recorded. Therefore, to encourage the children to attend to the visual stimuli, a short cartoon clip from a popular children’s movie, lasting approximately 3–4 s on average, was presented at 12 random intervals during the task. Participants were advised to listen to the auditory stimuli carefully, watch the visual stimuli carefully and press a button on a response box (“RTbox”; Li et al., 2010) whenever they saw a cartoon clip and as quickly as possible. A reward stars scale was used for further encouragement and to enable participants to track their progress. As each fifth of the experiment elapsed, an additional star was presented for 4 s until all five stars had been awarded at the end of the task. When the reward stars and visual stimuli were not displayed, a plain dark green background was shown with a small fixation cross positioned approximately in the same place as the speaker’s mouth on the screen, which participants were instructed to look at. A dark green background was chosen to match the color of the background in the visual speech condition clips (see Figure 1).

One complete run of the fNIRS imaging task lasted approximately 11 min. Depending on participant fatigue, the task was completed twice with a break in-between whenever possible. The cartoon clips used in the attentional trials differed in the second run to make the task more interesting. All of the NH participants and 18 CI users completed two runs of the fNIRS imaging task with only one CI user completing one run. To offer subjects an opportunity to become familiar with the task and stimuli, they completed a short practice session without wearing the fNIRS array. This was completed more than once if necessary.

### Stimuli

The Institute of Hearing Research Number Sentences (Hall et al., 2005) formed the speech stimuli for the fNIRS recordings. Ninety sentences recorded of a male speaker were available in both auditory and visual formats. For each fNIRS imaging run, the sentences were randomly allocated to form the four speech conditions and the silent condition. For the normal auditory speech condition, the auditory version of the sentence was presented with no additional processing. Similarly, for the visual speech condition, the visual element of the sentence was presented with no additional processing (i.e., a short video clip of a male speaker reciting the sentence but with no audio presented). A frame from one of the visual speech clips is displayed in Figure 1.

**Figure 2:**
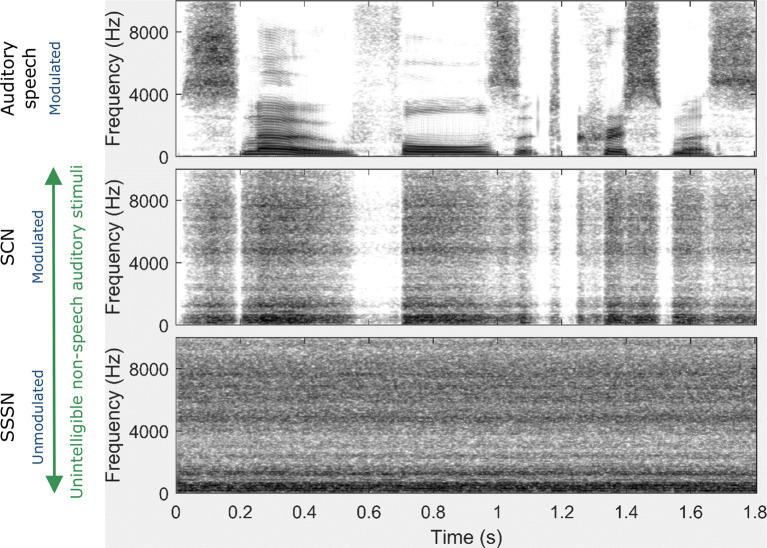
Spectrograms for each auditory stimulation condition for the sentence: Snow falls at Christmas. Unlike the auditory speech condition, there is a constant spread of energy across frequencies (indicated by constant levels of gray over time) in both the signal-correlated noise (SCN) and steady speech-shaped noise (SSSN) conditions. Although amplitude modulation (AM) is preserved in the SCN condition (as shown by the white gaps which mirror the gaps displayed in the auditory speech spectrogram), there is no AM in the SSSN condition.

To generate the SCN and SSSN conditions, a fast Fourier transform of the original speech signal was performed. The phase information was then randomized while the magnitude spectrum was preserved. This removed all of the temporal information in the original speech whilst retaining the energy distribution of across frequencies. The signal was then converted back to the time domain (forming SSSN) and, for the SCN condition only, was modulated by a low-pass (50 Hz) filtered envelope which was derived from the original sentence using the Hilbert transform. All speech stimuli were processed using MATLAB (Mathworks, Natick, MA, USA). Spectrograms of the same sentence for each auditory stimulation condition are displayed in Figure 2.

### Equipment

**Figure 3:**
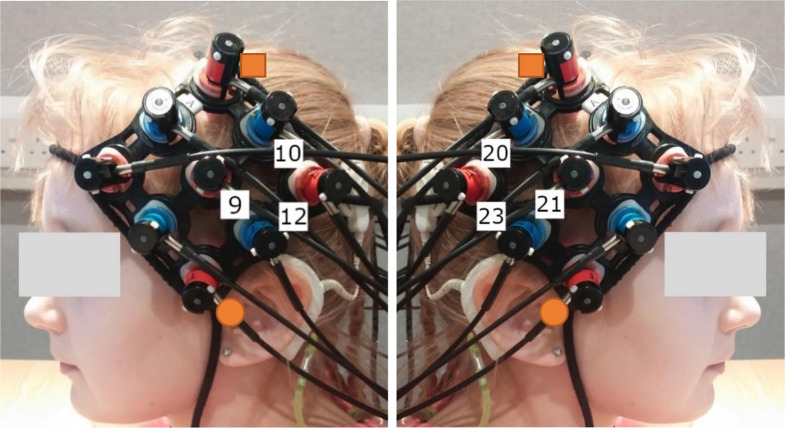
Typical optode array placement and approximate location of the channels forming the Region of Interest (ROI). The orange squares indicate point Cz and the orange circles indicate the preauricular point, as taken from the International 10-20 system (Jasper, 1958) to guide array placement. The ROI was selected to cover temporal brain regions bilaterally. Channels 9, 10, and 12 targeted the left hemisphere (LH) and channels 20, 21, and 23 targeted the right hemisphere (RH).

Subjects were comfortably seated approximately 75 cm away from a visual display monitor. Auditory stimuli were presented in the free-field from a Genelec 8030A loudspeaker mounted directly above the monitor at a level of 65 dB SPL (A-weighted root-mean-square level averaged for each sentence) measured at the subject’s listening position in their absence using a Brüel and Kjær Type 2250 sound level meter. The experiment was programmed in MATLAB using the Psychtoolbox-3 extensions (Brainard, 1997; Pelli, 1997; Kleiner et al., 2007). Brain activity was non-invasively measured using a continuous wave fNIRS system (ETG-4000, Hitachi Medical Company, Tokyo, Japan) at wavelengths of 695 nm and 830 nm (sampling rate 10 Hz), with crosstalk between channels and wavelengths minimized using frequency modulation (Scholkmann et al., 2014). Cortical activation was measured bilaterally using a pair 3 × 3 optode arrays with a fixed source-detector gap of 3 cm. Eighteen optodes were arranged in the arrays resulting in 12 measurement channels per hemisphere. Array placement over temporal brain regions was guided by the International 10-20 positioning system (Jasper, 1958) with efforts made to ensure positioning remained consistent between subjects as well as fNIRS runs. Specifically, the array was rotated to form a diamond arrangement with the lowest optode positioned as close to the preauricular point as possible, and the highest optode directed towards point Cz, as shown in Figure 3. If required, hair was moved from underneath optodes with a small illuminated tool to enhance optode-scalp contact. Importantly, although there was some variability in the position of the external CI processor between subjects in the CI group, the fNIRS array was always placed concurrently with the external components of the CI, with no optodes removed from the array and no array modifications or adaptations required for any subject. After the position of the array position had been finalized, a reference photograph was taken. During imaging, subjects were instructed to minimize unnecessary head movements and to keep as still to reduce motion artifacts in the fNIRS data. Testing was conducted within a sound-treated room with dimmed lighting.

### fNIRS Data Analysis

Analyses of fNIRS measurements were conducted in MATLAB in conjunction with functions from the HOMER2 package (Huppert et al., 2009) and custom scripts developed in our laboratory and previously used in our work (Dewey and Hartley, 2015; Wiggins et al., 2016; Anderson et al., 2017, 2019; Wijayasiri et al., 2017; Lawrence et al., 2018; Mushtaq et al., 2019). Channels with poor optode-scalp contact were removed using the scalp coupling index (SCI) technique by Pollonini et al. (2014). We excluded the worst 5% of channels (SCI threshold of ≥0.27) to preserve the maximum number of channels possible for statistical analyses, especially since the optode array did not permit spatially overlapping channels. Following this, raw fNIRS light intensity levels were converted into optical density using the HOMER2 *hmrIntensity2OD* function (Huppert et al., 2009). Motion artifact correction was applied using a wavelet filtering technique using the HOMER2 *hmrMotionCorrectionWavelet* function (Molavi and Dumont, 2012). Wavelet coefficients lying more than 0.719 times the interquartile range below the first or above the third quartiles were removed. Data were bandpass filtered between 0.02 and 0.5 Hz to attenuate cardiac oscillations and low-frequency drift and then converted into estimated changes of HbO and HbR concentration through the application of the modified Beer-Lambert law (Huppert et al., 2009). A default value of 6 was used for the differential path-length factor at both wavelengths. Note that the continuous-wave fNIRS system employed in this study cannot calculate absolute concentrations and only relative changes can be estimated, although this is sufficient for functional imaging experiments (Saliba et al., 2016). The functional hemeodynamic response was isolated using a signal separation algorithm by Yamada et al. (2012). This method assumes that changes in HbO and HbR concentrations are: (i) positively correlated in systemic physiological interference, which can then be suppressed; and (ii) negatively correlated in functional responses, which can then be isolated. We have previously shown that the implementation of this algorithm improves the reliability of fNIRS responses recorded from auditory brain regions (Wiggins et al., 2016). The response amplitude was quantified on a channel-wise basis using a general linear model approach (Schroeter et al., 2004). The design matrix consisted of a set of three regressors for each speech stimulus plus the silent condition, comprised of the canonical hemeodynamic response (provided in SPM8^1^) and its first two temporal derivatives (to capture responses with longer durations or which had moved in time; Friston et al., 1998; Lindquist and Wager, 2007; Lindquist et al., 2009; Wijayasiri et al., 2017). Each trial was modeled as a short epoch corresponding to the actual duration of stimulation for that trial. For each condition, the canonical and temporal-derivative regressors were serially-orthogonalized concerning one another (Calhoun et al., 2004). Two further sets of three regressors-of-no interest corresponding to the carton clips and progress stars were also included in the analysis to enable the model to incorporate all of the brain activity, even if not of interest. Model estimation was performed using a dual-stage ordinary least squares method described by Plichta et al. (2007) and serial correlation was corrected using the Cochrane and Orcutt’s (1949) method. The beta weights corresponding to the three regressors were then combined using the “derivative-boost” technique (Calhoun et al., 2004; Steffener et al., 2010) to enable estimated response amplitudes (ERAs) to be calculated. Note that the fNIRS data analysis procedure was conducted separately for each individual fNIRS run and the ERAs were then averaged across multiple runs from the same participant if appropriate.

### Region of Interest (ROI) Selection

**Figure 4:**
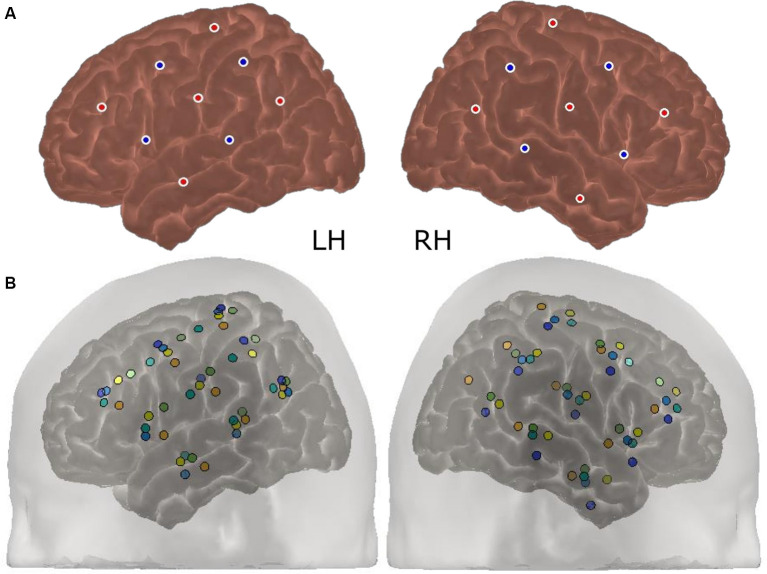
Average and individual optode positions of the array (*N* = 6). The average optode registration positions across six participants are shown in panel **(A)** for each cerebral hemisphere. The red dots represent fNIRS emitters and the blue dots represent detectors. Individual registrations, which are color-coded per participant, are displayed in panel **(B)**. The standard deviation in the position of each optode ranged between 5.7 and 11.6 mm.

ERAs corresponding to the channels forming the Region of Interest (ROI) were extracted for each condition (contrasted against silence). These were channels targeting auditory brain regions in the superior temporal cortex in both cerebral hemispheres, namely channels 9, 10, and 12 in the left hemisphere (LH) and channels 20, 21, and 23 in the right hemisphere (RH). The approximate location of these channels concerning the optode array is shown on a participant’s head in Figure 3. The ROI was defined using a combination of our laboratory’s previous work in adult CI listeners in which the same optode array was utilized (Anderson et al., 2017, 2019), and using average optode positions registered using a 3D digitizer. During this process, the position of the optodes and anatomical surface landmarks (left tragus, right tragus, nasion, inion, and Cz) were recorded and registered to the “Colin 27” atlas brain (Collins et al., 1998) using the AtlasViewer tool (Aasted et al., 2015). This enabled the location of the optodes relative to the surface landmarks to be calculated, which allowed optode positions, concerning underlying cortical anatomy, to be estimated. The average optode positions across six children for each hemisphere are shown in Figure 4A and individual registrations are shown in Figure 4B. The optode registration procedure confirmed that the same channels previously selected to form our adult ROI (Anderson et al., 2017, 2019) were also appropriate for the younger population involved in this experiment.

#### fNIRS Statistical Analyses

One-sided *t*-tests (α level 0.05) at a group-level (random-effects analysis) were used to test for statistically significant cortical activation. Each speech condition was contrasted against the silent baseline with a false discovery rate (FDR) correction (Benjamini and Hochberg, 1995) applied to control for multiple comparisons across fNIRS measurement channels. As well as conducting map-wise analyses over the whole optode array, ROI ERAs (single-subject level responses derived from the ROI for each condition) were used in a set of four linear mixed models (LMMs), one for each hypothesis. In all four LMMs, a random intercept for “participant” was included with “scaled identity” selected as the covariance type to account for the correlation between ERAs within a participant. A Restricted Maximum Likelihood estimation method was adopted. When investigating responses to visual speech and auditory speech, three fixed factors were included in each LMM (“group,” “hemisphere” and “group-hemisphere”). When examining cortical activation to intelligibility (auditory speech vs. SCN) and modulation (SCN vs. SSSN) the models included the same three factors as well as “condition,” “group-condition” and “condition-hemisphere.” Correlations between brain responses and speech perception scores were also performed. Analyses were conducted using IBM SPSS Statistics for Windows Version 26.0 software (IBM Corporation, Armonk, NY, USA).

## Results

### Speech Perception Test Results

**Figure 5:**
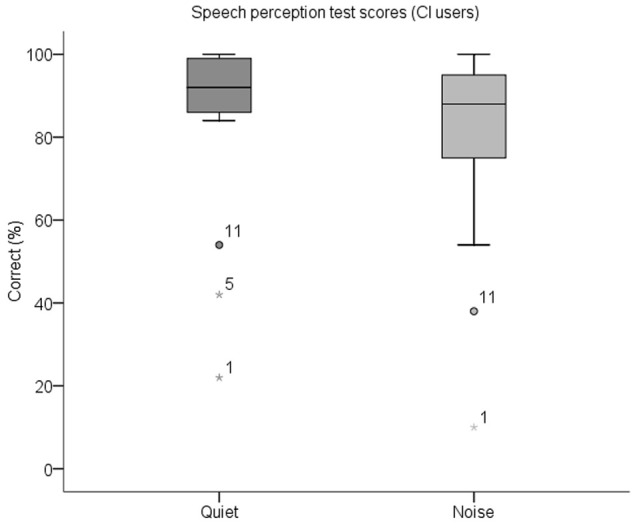
Box plots of speech perception scores in quiet and in noise for CI users. The median scorein quiet was 92% correct and in noise was 88% correct. CI participant IDs of outliers are also labeled.

A summary of the percentage of correctly identified keywords in both parts of the behavioral speech perception test by each CI user is shown in Table 3. Box plots of the same data are displayed in Figure 5. The median score for this group was 92% (IQR = 84–100) in quiet and 88% (IQR = 74–96) in noise. Subjects whose scores in either test were outliers (defined as falling beyond 1.5 × IQR below Q_1_ or above Q_3_) were deemed to be CI users with poor speech perception (*N* = 3), and the remaining CI users had good speech perception (*N* = 16). These three subjects are indicated with an asterisk in Table 3. All NH children scored 100% in both parts of the test except for one child who scored 98% in quiet and 96% in noise.

**Table 3:** Summary of each CI user’s speech perception test scores in quiet and in noise (*N* = 19).

Participant ID	Quiet (% correct)	Noise (% correct)
CI_01*	22	10
CI_02	88	74
CI_03	98	98
CI_04	94	90
CI_05*	42	54
CI_06	100	86
CI_07	98	96
CI_08	100	90
CI_09	100	98
CI_10	90	94
CI_11*	54	38
CI_12	88	76
CI_13	84	70
CI_14	84	84
CI_15	88	80
CI_16	100	100
CI_17	96	92
CI_18	92	88
CI_19	100	96
Mean	85	80

*The percentages of correctly identified keywords in quiet and in noise are displayed. Participant IDs marked with an asterisk correspond with CI users whose results in one or both parts of the test were outliers. These children were deemed to have poor speech perception*.

### Brain Imaging Results

#### Data Pre-processing

All 39 volunteers that participated, yielded usable fNIRS data. Note that in the case of one participant a system error towards the end of their second fNIRS run caused it to end prematurely. However, since over 90% of the fNIRS task had been completed, it was still included in the final analysis.

#### Contrasts Against Silence

**Figure 6:**
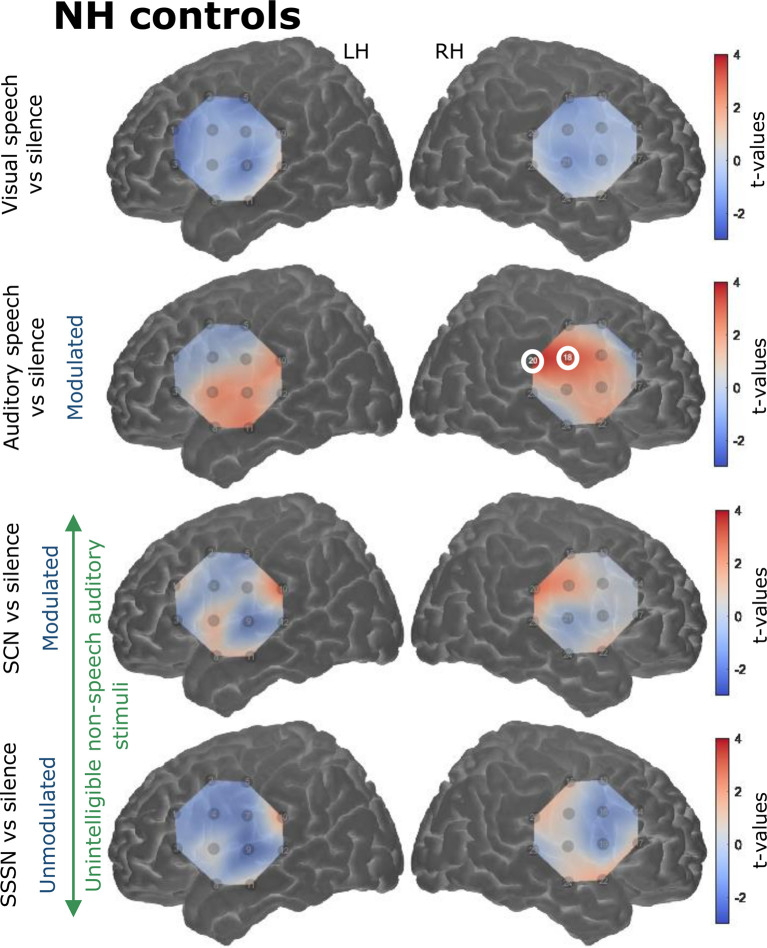
Group level cortical activation maps for each stimulation condition contrasted against silence in normally-hearing (NH) controls in the LH and the RH. Channels circled in white show statistically significant activation [*q* < 0.05, false discovery rate (FDR) corrected]. Only channels targeting temporal regions in the RH under the auditory speech condition were significantly activated. Note that the maps are interpolated from single-channel results and the overlay on the cortical surface is for illustrative purposes only.

**Figure 7:**
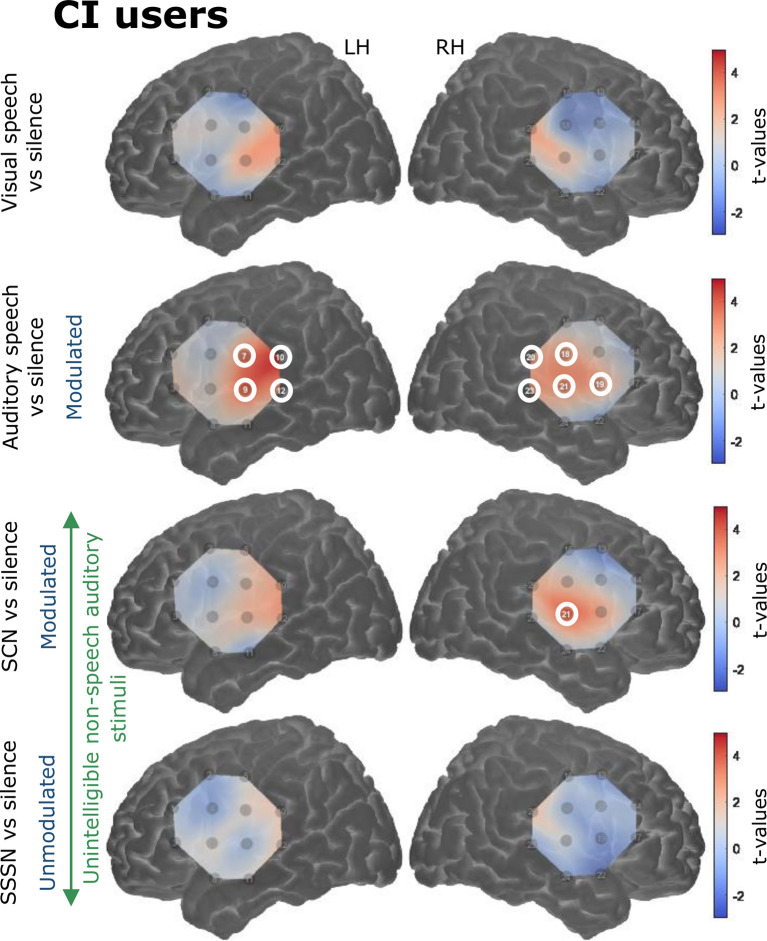
Group level cortical activation maps for each stimulation condition contrasted against silence in CI users in the LH and the RH. Channels circled in white show statistically significant activation (*q* < 0.05, FDR corrected). Channels targeting temporal regions in both hemispheres in response to auditory speech were significantly activated. Significant activation was also observed in right-sided temporal regions in response to signal-correlated noise (SCN). Note that the maps are interpolated from single-channel results and the overlay on the cortical surface is for illustrative purposes only.

Responses to the stimulation conditions were initially contrasted against the silent baseline. Group level activation maps for each condition contrasted against silence are shown in Figure 6 for the NH listeners and Figure 7 for the CI users. Statistically significant activation (*q* < 0.05, FDR corrected) was observed in temporal brain regions in response to auditory speech in both groups. This was limited to two channels in the RH in NH listeners, with a greater number of significantly activated channels observed in the CI group in both cerebral hemispheres. Four channels were significantly activated in the LH and five in the RH in the CI group. Additionally, statistically significant activation was also observed in the right temporal regions in response to SCN in the CI group only.

#### ROI Statistical Analyses: Between NH and CI Groups

**Figure 8:**
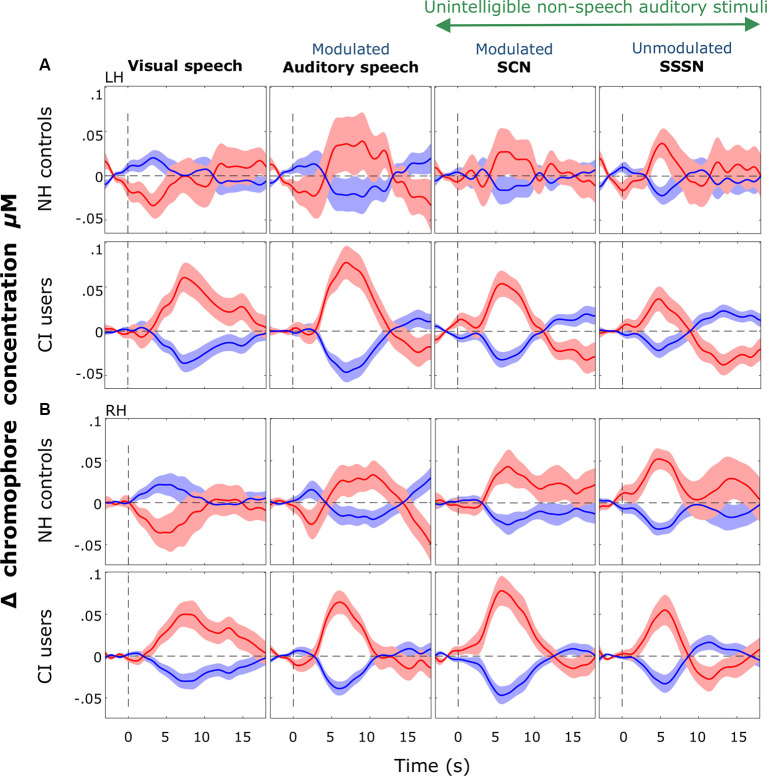
Block-averaged hemodynamic time courses derived from the ROI. These are displayed for each stimulation condition (response to silent trials subtracted out) for each group in the LH **(A)** and the RH **(B)**. Shaded regions indicate ±1 standard error of the mean across participants. Responses were averaged across channels 9, 10, and 12 in the LH and channels 20, 21, and 23 in the RH. Notably greater variation was observed in cortical responses recorded from the NH group than the CI group in both hemispheres but more so in the LH. Strong positive responses to visual speech were observed bilaterally in the CI group with a prominent deactivation shown in response to visual speech in NH controls.

Since most CI users attained good speech perception, we began by comparing cortical activation in NH controls with all deaf children with CIs, regardless of their speech perception scores, using LMMs. Subsequent analysis correlated cortical responses with speech performance within the CI group. Block-averaged hemodynamic time courses derived from the ROI channels in the LH and the RH for both groups are shown in Figure 8.

**Figure 9:**
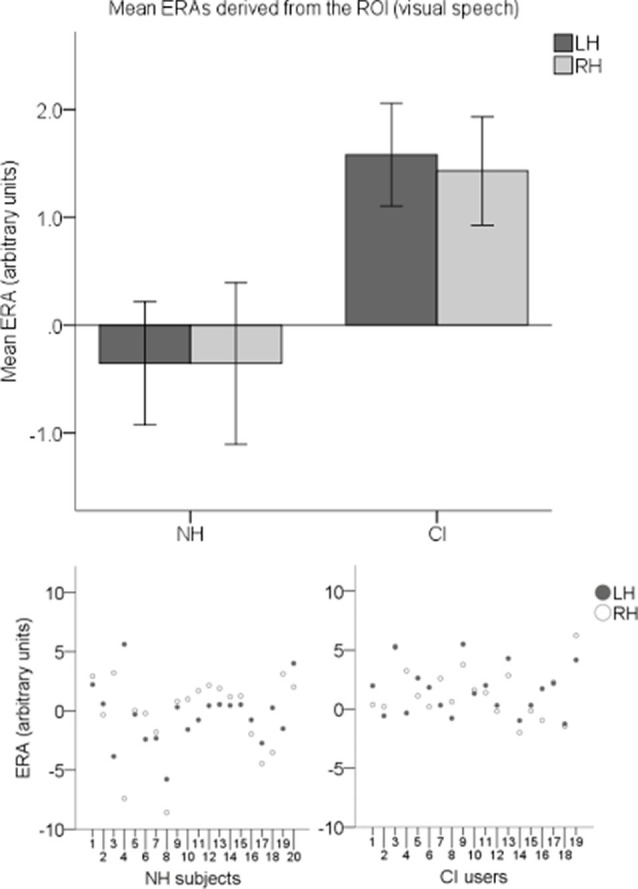
Individual estimated response amplitudes (ERAs) and group level means (*N* = 39) derived from the ROI for the visual speech condition. The bar chart shows mean ERAs for each group corresponding to channels 9, 10, and 12 in the LH and channels 20, 21, and 23 in the RH. Error bars show ±1 standard error of the mean. Scatterplots showing individual ERA values are displayed underneath for each group.

ERAs were calculated to conduct statistical analyses. To investigate responses to visual speech, an LH and an RH ERA value (derived from the ROI) for each participant for the visual speech condition were entered into an LMM. Individual ERAs and group level means for each hemisphere are shown in Figure 9 for this condition. No statistically significant main effects of hemisphere (*F*_(1,37)_ = 0.024, *p* = 0.878) or group-hemisphere interaction (*F*_(1,37)_ = 0.024, *p* = 0.878) were found. However, there was a statistically significant main effect of group (*F*_(1,37)_ = 7.542, *p* = 0.009) with responses to visual speech larger in CI users than NH controls (mean difference = 1.862, 95% CI: (0.488, 3.236) arbitrary units).

**Figure 10:**
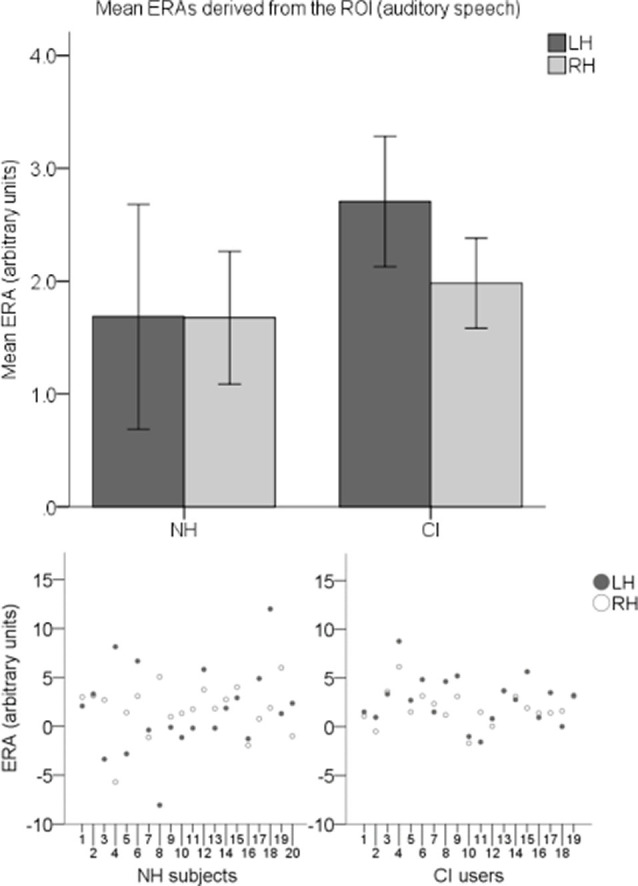
Individual ERAs and group level means (*N* = 39) derived from the ROI for the auditory speech condition. The bar chart shows mean ERAs for each group corresponding to channels 9, 10, and 12 in the LH and channels 20, 21, and 23 in the RH. Error bars show ±1 standard error of the mean. Scatterplots showing individual ERA values are displayed underneath for each group.

To explore cortical activation to auditory speech, an LH and an RH ERA value (derived from the ROI) for each participant for the auditory speech condition were entered into a second LMM. Individual ERAs and group level means for each hemisphere are shown in Figure 10 for this condition. No statistically significant main effects of group (*F*_(1,37)_ = 0.914, *p* = 0.345) or hemisphere (*F*_(1,37)_ = 0.294, *p* = 0.591) were found. There was also no statistically significant interaction between the two (*F*_(1,37)_ = 0.279, *p* = 0.6).

**Figure 11:**
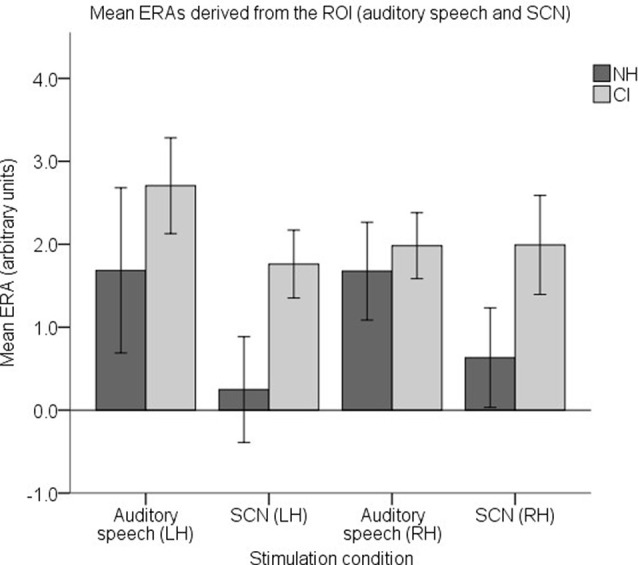
Mean ERAs (*N* = 39) derived from the ROI for the auditory speech and SCN conditions. Mean ERAs are shown for each group corresponding to channels 9, 10, and 12 in the LH and channels 20, 21, and 23 in the RH. Error bars show ±1 standard error of the mean.

To examine responses to speech intelligibility, a set of four ROI ERAs per participant were entered into another LMM, this time corresponding to the LH and RH under the auditory speech and SCN conditions. Mean group level ERAs are displayed in Figure 11. No statistically significant main effects of group (*F*_(1,37)_ = 2.842, *p* = 0.1) or hemisphere (*F*_(1,112)_ = 0.006, *p* = 0.937) were observed. Again, no statistically significant group-condition (*F*_(1,112)_ = 1.104, *p* = 0.296), group-hemisphere (*F*_(1,112)_ = 0.35, *p* = 0.555) or condition-hemisphere (*F*_(1,112)_ = 0.826, *p* = 0.365) interaction was found. However, there was a statistically significant main effect of condition (*F*_(1,112)_ = 5.405, *p* = 0.022) with responses to auditory speech larger than responses to SCN (mean difference = 0.854, 95% CI: (0.126, 1.581) arbitrary units).

**Figure 12:**
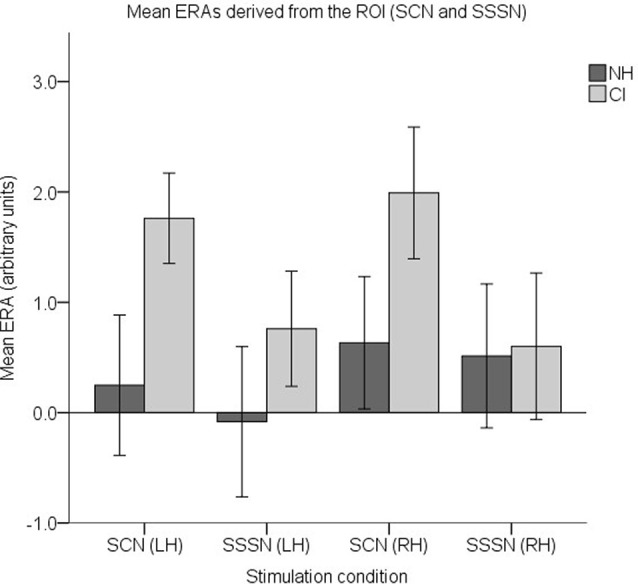
Mean ERAs (*N* = 39) derived from the ROI for the SCN and SSSN conditions. Mean ERAs are shown for each group corresponding to channels 9, 10, and 12 in the LH and channels 20, 21, and 23 in the RH. Error bars show ±1 standard error of the mean.

Finally, to examine responses to AM, a set of four ROI ERAs per participant (corresponding to the LH and RH under the SCN and SSSN conditions) were entered into a fourth LMM. Group level ERA means are displayed in Figure 12. Similar to the previous analysis, no statistically significant main effects of group (*F*_(1,37)_ = 2.508, *p* = 0.122) or hemisphere (*F*_(1,112)_ = 0.565, *p* = 0.454) were found. There was also no statistically significant interaction between group and condition (*F*_(1,112)_ = 1.927, *p* = 0.168), group and hemisphere (*F*_(1,112)_ = 0.424, *p* = 0.516) or condition and hemisphere (*F*_(1,112)_ = 0.014, *p* = 0.906). However, there was a statistically significant main effect of condition (*F*_(1,112)_ = 4.129, *p* = 0.045) with responses to SCN larger than responses to SSSN (mean difference = 0.710, 95% CI: (0.018, 1.403) arbitrary units).

#### ROI Statistical Analyses: Within the CI Group

Since only three of the nineteen CI users scored poorly on the speech perception test, it was not possible to conduct formal statistical analyses between CI users with good vs. poor speech perception due to a lack of statistical power. Therefore, correlations were performed instead between bilateral ROI ERAs contrasted against silence and speech perception scores (percentage of keywords correctly identified) in noise (as scores in quiet were more prone to ceiling effects) to assess the direction of any relationships.

A statistically significant correlation was not observed between speech perception scores and visual speech ERAs or (*τ*b = 0.236, *p* = 0.161) or auditory speech ERAs (*τ*b = 0.189, *p* = 0.262). To investigate the effect of intelligibility, SCN ERAs were subtracted from auditory speech ERAs. Again, no statistically significant correlation was observed (*τ*b = −0.047, *p* = 0.779) when the differences were correlated against speech perception scores. Finally, to examine modulation effects, SSSN ERAs were subtracted from SCN ERAs with the differences then correlated against speech scores. Once again, no statistically significant correlation was observed (*τ*b = −0.142, *p* = 0.4).

## Discussion

Previous work in deaf adults has demonstrated that fNIRS responses are related to behavioral speech performance following cochlear implantation (e.g., Chen et al., 2016; Olds et al., 2016; Anderson et al., 2017, 2019; Zhou et al., 2018). We aimed to extend these findings to a pediatric sample of NH and CI listeners. We examined the relationship between speech performance and cortical fNIRS responses and found that CI users process auditory, but not visual input, similarly to NH children.

Consistent with our hypothesis, we observed significantly greater activation in response to visual stimuli in CI users compared with NH listeners. To our knowledge, this is the first time this cross-modal activation has been shown in response to visual speech in pediatric CI users with primarily congenital and pre-lingual deafness. Although Campbell and Sharma (2016), in their EEG study, found evidence of cross-modal recruitment in children with CIs, this was in response to visual gratings; a low-level non-speech stimulus very different from the visual speech stimuli used in the present work. Furthermore, the authors found a similar direction of activation in both CI users and NH controls (Campbell and Sharma, 2016), unlike the clear deactivation we found in NH listeners in response to visual speech (as shown in the hemodynamic time courses in Figure 8).

The positive influence of a synergistic audiovisual speech perception mechanism on hearing restoration has previously been observed in post-lingually deaf adults (e.g., Sandmann et al., 2012; Anderson et al., 2017), which is logical since this population has previous experience of using visual cues when listening to speech which can be made use of once hearing is restored. However, in the case of pre-lingual deafness, no such previous experience of audiovisual integration during speech listening exists for listeners to refer back to. Additionally, given the well-established (human and animal) literature that indicates early exposure to sound is vital for proper development of the auditory system (Kral et al., 2000; Sharma et al., 2002; Kral and Eggermont, 2007; Kral, 2009, 2013), it is reasonable to presume that activation of auditory regions by visual stimuli is a reflection of poor early auditory development, which would, in turn, be linked with poor outcomes. However, even in our primarily pre-lingually deafened group of school-age CI listeners, the majority of whom had good speech perception, we still observed strong responses to visual speech in auditory brain regions, which was not found in NH listeners. Contrary to our findings, it could be hypothesized that pediatric CI listeners with good speech performance would not exhibit such strong responses to visual speech in auditory regions (i.e., they would demonstrate a more “normal” response). However, our findings show that cross-modal plasticity is adaptive for the successful development of auditory speech perception following cochlear implantation, regardless of whether or not an individual has previous audiovisual language experience.

Alternatively, the visual activation we observed in auditory brain regions in the CI listeners perhaps does not influence the responsiveness of their auditory cortices and their auditory processing remains unaltered (Land et al., 2016). Put simply, the cross-modal reorganization is neither adaptive nor maladaptive; it is correlated but not causally related. This could be a consequence of auditory deprivation in the past. Perhaps neurons within a deprived auditory cortex lack inhibition such that any stimulation causes excitation regardless of the stimulus modality (Butler and Lomber, 2013). This potential justification for the results observed cannot be definitively explored without sufficient power to statistically compare cortical responses in CI listeners with good and poor speech perception, which the current study did not have. However, this explanation remains unlikely as the close relationship between cortical reorganization and CI outcomes is well established (Kral and Sharma, 2012; Kral, 2013). For now, it would appear that a synergistic relationship exists between cortical responsiveness to visual speech and auditory speech perception in children with CIs, a result consistent with adult fNIRS work previously conducted in our laboratory (Anderson et al., 2017).

Importantly, no statistically significant differences were observed between the two groups in response to auditory speech or the two auditory contrasts of interest (auditory speech vs. SCN and SCN vs. SSSN). In all participants overall, greater activation was exhibited in response to intelligible speech than unintelligible sounds, and modulated than unmodulated auditory signals, with no effects of group. Similar to previous adult fNIRS work conducted by Olds et al. (2016), in which responses to intelligible vs. unintelligible stimuli in NH listeners and good CI users were comparable, these findings indicate that functional brain patterns to auditory stimuli recorded using fNIRS in NH children and good CI listeners overlap considerably. This suggests that pediatric CI recipients can learn to process auditory input with similar outcomes as controls, even with a highly modified auditory periphery. It further demonstrates that CI listeners have the fundamental functional ability to decipher between auditory inputs that contain characteristics of normal speech (e.g., intelligibility and AMs) from those that do not. However, it is not possible to unravel the factors contributing towards this ability (e.g., optimal CI programming or enhanced cognitive abilities) from the present paradigm.

When compiled together, our results reveal that any auditory processing deficit arising following the cross-modal reorganization of the auditory cortex is not as detrimental as previously thought and the heightened sensitivity observed in response to visual speech does not appear to be at the expense of sensitivity to auditory stimuli. The visual modality may help to *fill in the gaps* when speech is degraded (Rouger et al., 2007; Strelnikov et al., 2013). Perhaps through cortical reorganization, deaf individuals generally become better multisensory integrators as a compensation mechanism and develop an enhanced ability to connect visual and auditory inputs (Rouger et al., 2007). Alternatively, since the majority of CI subjects were good performers, it is also possible that these individuals were able to ignore the visual input and process auditory information well, especially since a statistically significant correlation was not identified between speech perception scores in noise and fNIRS responses to either visual speech. However, this is unlikely given a null result was equally observed between behavioral speech scores and responses to auditory speech.

It has been argued that a sensitive period for auditory processing should be considered in parallel with a sensitive period for language processing (Lyness et al., 2013). Therefore, the strong responsiveness to visual speech observed in good CI performers may be a reflection of better visual language development during the sensitive period for language learning (Lyness et al., 2013). Given the fact that vision is the main modality by which deaf children access language before implantation, strong visual responses may reflect strong cortical language processing circuitry (Lyness et al., 2013). These strengthened visual language networks may provide a means for understanding auditory signals *via* an audiovisual mechanism, translating into better speech performance (Lyness et al., 2013).

Our findings have important implications on the post-implantation rehabilitation strategies offered to pediatric CI recipients (Strelnikov et al., 2009; Woodhouse et al., 2009; Lyness et al., 2013). Traditional post-operative interventions do not typically reinforce the use of visual information (e.g., speech reading) and, rather, discourage it for fear of disrupting auditory processing of auditory speech (Woodhouse et al., 2009; Lyness et al., 2013). This may be a consequence of prolonged clinical and research focus on the visual takeover of the auditory cortex revealed by numerous animal models, which focus solely on the development of the auditory system and auditory processing, and not on language processing and linguistic development (Lyness et al., 2013). With attention primarily focused on ensuring the auditory cortex remains sensitive to auditory input, the multimodal nature of speech processing is often overlooked (Lyness et al., 2013). Our results demonstrate that there is not necessarily a trade-off between visual vs. auditory processing in auditory cortices of CI recipients with good speech performance. Consequently, rehabilitation programs should consider exploiting the pre-operative visual language development CI listeners undergo for post-operative auditory signal interpretation by encouraging the integration of both the auditory and visual modalities and, perhaps, direct attention towards speech reading abilities (Schwartz et al., 2004; Strelnikov et al., 2015; Anderson et al., 2017).

Although our results indicate that successful speech perception may (at least partially) be due to the ability to combine auditory and visual inputs in a cohesive manner (Rouger et al., 2007), the mechanisms underlying the cross-modal plasticity detected in CI listeners are not well known and there is speculation regarding the factors that may be contributing towards this observation. For example, it has been suggested that visual input supports sound localizing abilities following hearing restoration (Akeroyd, 2014; Isaiah et al., 2014), or is integrated with auditory input to maintain speech recognition in challenging listening environments (Strelnikov et al., 2009). Alternatively, vision may be deemed to be the most reliable sensory channel for CI users so is favored when the incoming auditory signal is ambiguous (Strelnikov et al., 2009), or visual information offers top-down guidance for auditory perceptual learning (Bernstein et al., 2013). It is likely that neuroplasticity, due to its complexity, is a result of a combination of all of these factors and others. Nonetheless, regardless of the mechanisms driving cortical plasticity and responsiveness, it is apparent that the effects of cortical reorganization are intricate and worthy of further exploration. Future work should identify whether some pediatric CI users develop better speech skills due to an innate ability to combine visual information with auditory input from birth or whether this is a skill that develops over time and with experience in good CI listeners only. It would also be beneficial to investigate whether patterns are speech-specific responses (i.e., visual speech) or whether CI users respond more strongly to all visual stimuli (i.e., visual non-speech).

An important caveat to note is although responses to both auditory and visual speech were recorded in the current paradigm, this was done so separately for each modality. The interaction between the two modalities, the role of audiovisual integration, as well as any dependency of these factors on speech perception, was not explicitly tested. Therefore, even with the strong activation elicited by visual speech observed exclusively in CI listeners, and the profound deactivation elicited in NH controls in response to the same stimulus, the suggestion that high speech recognition levels in CI recipients are a direct result of the use of visual input or the consequence of bimodal integration or interaction is not definitive. It is possible that the auditory and visual modalities in these subjects do not strongly interact or interfere at all, and that cross-modal plasticity has only a minimal influence on sensory restoration, a phenomenon supported by some animal models (Land et al., 2016).

As all but three CI users performed very well in the speech perception test, it was not possible to contrast CI listeners with good speech scores against those with poorer scores. Perhaps the lower recruitment figure of poor CI users reflects the proportion of good vs. poor CI performers in the United Kingdom. Alternatively, it may be a consequence of (parents of) better performers being more inclined towards engaging in research activities. Undoubtedly, future work must involve CI users with a more diverse range of speech abilities. Although correlations were performed within the CI group, no statistically significant effects were found. This was unsurprising given the homogeneity of the majority of the speech performance data.

Interestingly, group-level cortical activation maps (displayed in Figures 6, 7) showed a greater number of significantly activated channels in CI users compared with controls in response to auditory speech. Evidence from animal models indicates that, following deafness, heightened excitability is observed in the auditory cortex, which may be a reflection of the brain’s attempt to ensure that a functioning and operational level of excitability is sustained (Kotak et al., 2005). Therefore, perhaps the CI users in this study demonstrated greater activation compared to NH children, in terms of cortical area, because of this heightened excitability. Furthermore, previous neuroimaging research has shown that speech elicits activation in more cortical regions in CI listeners compared to NH controls (e.g., Naito et al., 2000). This may be because a greater neuronal activity is required in CI users to decode speech signals coded by a CI in comparison to neuronal activation required by NH listeners to decode normal speech signals (Naito et al., 2000). Additionally, the smaller area of statistically significant activation observed in NH could be a reflection of a more tightly packed and specialized region for (normal) speech-specific processing. This may be lacking in CI users, perhaps due to the influence of early auditory deprivation on brain structure and function (Shepherd et al., 1997; Kral et al., 2000; Klinke et al., 2001; Klinke, 2008; Gordon et al., 2011). Also, substantially more variation was displayed in the NH group’s hemodynamic time courses, as illustrated in Figure 8, whereas the responses elicited by the CI users were more uniform (which may also be a result of increased excitability in the primary auditory cortex). The greater variability and excessive noise in the control group will likely have damped the clarity of the measurements and perhaps contributed to some of the null results observed. It is also worth noting that one channel was significantly activated in response to SCN in the right temporal regions in the CI group only (see Figure 7). Again, it is possible that greater response variability in NH listeners contributed to the null result in this group. The modulating envelope of the SCN stimulus seemed to activate regions involved in speech processing, whereas the steady-state envelope of the SSSN condition did not. Whilst it remains unclear as to why significant activation was limited to the RH, fNIRS studies have previously reported that the RH can be more responsive to speech signals, compared with the contralateral side (Pollonini et al., 2014).

In conclusion, school-age CI users positively utilize cross-modal recruitment of auditory brain regions by visual speech to enhance their speech perception, a phenomenon not observed in NH listeners. Like children with normal hearing, pediatric CI users also show stronger cortical fNIRS responses to intelligible speech and modulated signals, compared with unintelligible sounds and unmodulated noise, respectively. These neuroimaging findings could help form the basis for a clinical measure of pediatric CI outcome, and imply that post-operative rehabilitation strategies for children should include visual information.

## Data Availability Statement

The raw data supporting the conclusions of this article will be made available by the authors, without undue reservation.

## Ethics Statement

The studies involving human participants were reviewed and approved by the East Midlands—Leicester Central Research Ethics Committee and the University of Nottingham Faculty of Medicine & Health Sciences Research Ethics Committee. Written informed consent to participate in this study was provided by the participants’ legal guardian/next of kin. Written informed consent was obtained from the minor(s)’ legal guardian/next of kin for the publication of any potentially identifiable images or data included in this article.

## Author Contributions

FM, IW, PK, and DH were involved in the conception and design of the study. FM recruited participants and collected the data. FM, IW, and PK analyzed the data. FM wrote the manuscript. IW, CA, and DH commented on and reviewed the manuscript.

## Conflict of Interest

The authors declare that the research was conducted in the absence of any commercial or financial relationships that could be construed as a potential conflict of interest.

## Footnotes

^1^http://www.fil.ion.ucl.ac.uk/spm
